# Hydrogen Peroxide Production by the Spot-Like Mode Action of Bisphenol A

**DOI:** 10.3389/fpls.2020.01196

**Published:** 2020-08-05

**Authors:** Ioannis-Dimosthenis S. Adamakis, Ilektra Sperdouli, Eleftherios P. Eleftheriou, Michael Moustakas

**Affiliations:** ^1^ Department of Botany, Faculty of Biology, National and Kapodistrian University of Athens, Athens, Greece; ^2^ Institute of Plant Breeding and Genetic Resources, Hellenic Agricultural Organization Demeter, Thessaloniki, Greece; ^3^ Department of Botany, Aristotle University of Thessaloniki, Thessaloniki, Greece

**Keywords:** bisphenol A, chlorophyll fluorescence imaging, plastoquinone pool, signaling molecule, H_2_O_2_ scavenger, photosystem II functionality, necrotic death-like spot, reactive oxygen species

## Abstract

Bisphenol A (BPA), an intermediate chemical used for synthesizing polycarbonate plastics, has now become a wide spread organic pollutant. It percolates from a variety of sources, and plants are among the first organisms to encounter, absorb, and metabolize it, while its toxic effects are not yet fully known. Therefore, we experimentally studied the effects of aqueous BPA solutions (50 and 100 mg L^−1^, for 6, 12, and 24 h) on photosystem II (PSII) functionality and evaluated the role of reactive oxygen species (ROS) on detached leaves of the model plant *Arabidopsis thaliana*. Chlorophyll fluorescence imaging analysis revealed a spatiotemporal heterogeneity in the quantum yields of light energy partitioning at PSII in *Arabidopsis* leaves exposed to BPA. Under low light PSII function was negatively influenced only at the spot-affected BPA zone in a dose- and time-dependent manner, while at the whole leaf only the maximum photochemical efficiency (F*v*/F*m*) was negatively affected. However, under high light all PSII photosynthetic parameters measured were negatively affected by BPA application, in a time-dependent manner. The affected leaf areas by the spot-like mode of BPA action showed reduced chlorophyll autofluorescence and increased accumulation of hydrogen peroxide (H_2_O_2_). When H_2_O_2_ was scavenged *via* N-acetylcysteine under BPA exposure, PSII functionality was suspended, while H_2_O_2_ scavenging under non-stress had more detrimental effects on PSII function than BPA alone. It can be concluded that the necrotic death-like spots under BPA exposure could be due to ROS accumulation, but also H_2_O_2_ generation seems to play a role in the leaf response against BPA-related stress conditions.

## Introduction

Plants are sessile organisms, specially affected by changes in their environment and therefore unavoidably prone to many stress-factors. So, plants have evolved an extensive range of mechanisms for acclimation and adaptation (van Loon, 2016). Numerous studies have confirmed that some of these mechanisms include reactive oxygen species (ROS) formation (Garg and Manchanda, 2009; Foyer, 2018; Huang et al., 2019). These molecules were traditionally related to wide-range damaging of cellular macromolecules (i.e., nucleic acids, lipids, proteins, etc.), which probably could result in cell death and even whole organism collapse (Potters et al., 2010). Nonetheless, decades of thorough research gathered substantial evidence to support that ROS-mediated responses are orchestrated and regulated under a tight genetic control. Hence, in plants, ROS roles in early signaling events initiated by various environmental stimuli have been established (Noctor et al., 2018; Huang et al., 2019). These stimuli could include extreme temperatures (Awasthi et al., 2015), drought (Laxa et al., 2019), heavy metals (Eleftheriou et al., 2015), nanoparticle**s** (Sperdouli et al., 2019), and organic pollutant**s** (Christou et al., 2018).

One such organic pollutant is bisphenol A (2,2-bis(4-hydroxyphenyl)propane; BPA), a chemical stabilizer widely applied in the industrial manufacture of plastic materials (Lin et al., 2017). As plastic commodities deteriorate, BPA can escape and pollute the environment (Xu et al., 2011). This pollution seems to be harmful, since, BPA belongs to the xenoestrogen substance family and by acting as an endocrine disruptor can cause several human health issues (Jalal et al., 2018; Abraham and Chakraborty, 2019). While extensive research has been conducted about BPA effects on humans/animals, scientific data regarding the toxic effects of BPA on plants have been accumulating only in recent years (Xiao et al., 2020). Although plants can absorb and metabolize BPA, at the same time BPA could deteriorate their cellular/physiological status (Zhang et al., 2017). It has been shown that experimentally applied concentrations of BPA (mg/L) negatively affected the growth of many important crops, e.g., soybean (Qui et al., 2013; Zhang et al., 2016; Jiao et al., 2017; Li X. et al., 2018; Zhang et al., 2018; Xiao et al., 2019), pea (Adamakis et al., 2013), wheat (Adamakis et al., 2019), maize (Stavropoulou et al., 2018), rice (Ali et al., 2016), cucumber (Li Y. T. et al., 2018) and onion (Adamakis et al., 2019); also of non-cultivated plants such as the Cephalonian fir (Adamakis et al., 2016) and the model plant *Arabidopsis thaliana* (Pan et al., 2013; Tian et al., 2014; Frejd et al., 2016; Ali et al., 2017; Rapala et al., 2017; Bahmani et al., 2020). Growth reduction effects have interestingly been found to occur also after environmentally relevant concentrations (μg/L) applied on cultivated crops, e.g., cabbage and tomato (Staples et al., 2010), native plants such as oat (Staples et al., 2010) and seagrasses (Adamakis et al., 2018; Malea et al., 2020).

BPA-derived growth defects have been linked to either cytoskeletal derangement (Adamakis et al., 2013; Adamakis et al., 2016; Adamakis et al., 2018; Stavropoulou et al., 2018; Adamakis et al., 2019), hormonal imbalance (Frejd et al., 2016; Li X. et al., 2018; Bahmani et al., 2020), deterioration of the photosynthetic machinery (Jiao et al., 2017; Kim et al., 2018; Li Y. T. et al., 2018) or ROS production (Wang et al., 2015; Ali et al., 2016; Zhang et al., 2018; Xiao et al., 2019). It could therefore be concluded that BPA effects in plants are pleiotropic (Xiao et al., 2020). However, the increased demand for BPA and focus on BPA research over the past years (Shafei et al., 2018), has gathered significant amount of evidence indicating that the induction of ROS is the start of a cascade of BPA-induced cellular effects. As such, ROS contribute significantly to BPA toxic and carcinogenic potential (Moura et al., 2010). Specifically for plants, BPA effects on photosynthesis have been linked to ROS production (Li Y. T. et al., 2018), but fascinatingly a protective role for ROS in the plant response against BPA has been also proposed (Zhang et al., 2018), a phenomenon also observed in animal models (Guo et al., 2017; Durovcova et al., 2018) under BPA exposure.

It is evident that any change or imbalance in the function of the chloroplast will affect directly or/and indirectly the other cellular functions of the plant cell (Bobik and Burch-Smith, 2015). Earlier studies have suggested that the redox state of the plastoquinone (PQ) pool initiates plant acclimation and is of unique significance for antioxidant defense and signaling (Hüner et al., 2012). Consequently, it can be hypothesized that BPA stress in plants, like in animals (Moura et al., 2010), could be initially sensed *via* ROS-production; then the associated changes in the chloroplast oxidoreduction homeostasis synergistically with other signaling pathways could induce physiological or/and molecular adaptive responses. In order to test this hypothesis and provide novel insights into mechanisms of BPA effects to plant physiological functions such as photosynthesis, we experimentally studied the effects of BPA aqueous solutions on several parameters of photosystem II (PSII) functionality in detached leaves of the model plant *Arabidopsis thaliana*. In particular, we investigated whether the BPA-induced hydrogen peroxide (H_2_O_2_) in combination with the H_2_O_2_ scavenger, N-acetylcysteine, has a positive or negative action on the selected photosynthetic parameters.

## Materials and Methods

### Plant Material and Growth Conditions

*Arabidopsis thaliana* (L.) Heynh. (Col-0) seeds, obtained from Nottingham Arabidopsis Stock Centre (NASC), were bleach surface sterilized and after being imbibed at 4°C for 24 h were sown directly on soil. Emerged seedlings were left to grow at a 22 ± 1°C temperature and a 16-h/8-h light/dark cycle at 120 µmol photons m^−2^ s^−1^ light intensity and 60 ± 5% day/night humidity for 4 weeks. Rosette leaves 8 from 4-week-old plants were cut and further on processed.

### BPA and NAC Treatments

Detached leaves of *A. thaliana* maintained in Petri dishes on filter paper soaked with distilled water were considered as controls. Four to five leaves per experiment were treated with aqueous 50 and 100 mg L^−1^ (0.2 and 0.4 mM) BPA solutions, prepared from a stock solution of 200 mg L^−1^ at 21.5°C, pH 7.0 (Staples et al., 1998; Adamakis et al., 2013; Adamakis et al., 2019), soaked on filter paper in Petri dishes, for 6, 12 and 24 h. Each treatment has been done in triplicate.

N-acetylcysteine (NAC) is a ROS scavenger capable of interacting with H_2_O_2_ (Aruoma et al., 1989; Zafarullah et al., 2003; Ezeriņa et al., 2018). We applied NAC on detached *A. thaliana* leaves to evaluate the result of H_2_O_2_ scavenging in combination with BPA action. Leaves were treated with either 500 µM NAC (Muranaka et al., 2013; Livanos et al., 2016; Colak et al., 2019) or with 50 mg L^−1^ (0.2 mM) BPA plus 500 µM NAC or with 50 mg L^−1^ BPA alone for 24 h. All treatments were performed with three independent biological replicates.

### Hydrogen Peroxide Imaging Detection

H_2_O_2_ detection in *A. thaliana* leaves was implemented as described earlier (Moustaka et al., 2015). Briefly, leaves were incubated for 30 min with 25 µM 2′, 7′-dichlorofluorescein diacetate (H_2_DCF-DA, Sigma) in 10 mM Tris-HCl (pH 7.4) in dark. The leaves were observed under a Zeiss AxioImager.Z2 fluorescence microscope at excitation and emission wavelengths of 480 and 530 nm, respectively (Moustaka et al., 2015). An AxioCam MRc 5 camera attached to the microscope captured the images. Autofluorescence signal interference was also checked (Moustaka et al., 2018). All treatments were performed with three independent biological replicates.

### Chlorophyll Fluorescence Imaging Analysis

A modulated chlorophyll fluorescence system (Imaging PAM M-Series system, Heinz Walz Instruments, Effeltrich, Germany) was used to evaluate the spatiotemporal effects of BPA on PSII photochemistry. Chlorophyll fluorescence in dark-adapted (for 20 min) detached *A. thaliana* leaves was measured at room temperature as described previously (Moustaka et al., 2015). Two light intensities were used for chlorophyll fluorescence measurements, a low light intensity (140 μmol photons m^−2^ s^−1^) that was similar to the growth light and a high light intensity (1000 μmol photons m^−2^ s^−1^). Color-coded images are presented of dark adapted leaves of (a) the maximum photochemical efficiency (F*v*/F*m*), and after 5 min of illumination, (b) the effective quantum yield of PSII photochemistry (Φ_PSII_) that estimates the efficiency by which light absorbed by PSII is used for photochemistry, (c) the quantum yield of regulated non-photochemical energy loss in PSII (Φ_NPQ_), (d) the quantum yield of non-regulated energy loss in PSII (Φ_NO_), and (e) the photochemical quenching (q_p_), a measure of the fraction of open PSII reaction centers, that is the redox state of the plastoquinone (PQ) pool. Nine to fourteen areas of interest (AOIs) were selected in each leaf so as to have representative areas of the whole leaf.

### Statistical Analyses

Statistically significant differences were evaluated for the chlorophyll fluorescence parameters of Control Whole Leaves (CWL), BPA treated Whole Leaves (BWL), Spot BPA zone (SPB), Spot Surrounding Area (SSA) and the Rest of the Leaf (RL), that is the leaf area that remains if the Spot BPA zone (SPB) and the Spot Surrounding Area (SSA) are subtracted from the BPA-treated Whole Leaves (BWL). The measured chlorophyll fluorescence parameters were analyzed by t-test at a level of P < 0.05 (StatView computer package, Abacus Concepts, Inc Berkley, CA, USA). Data are presented as means from three independent experiments.

## Results

**Figure 1:**
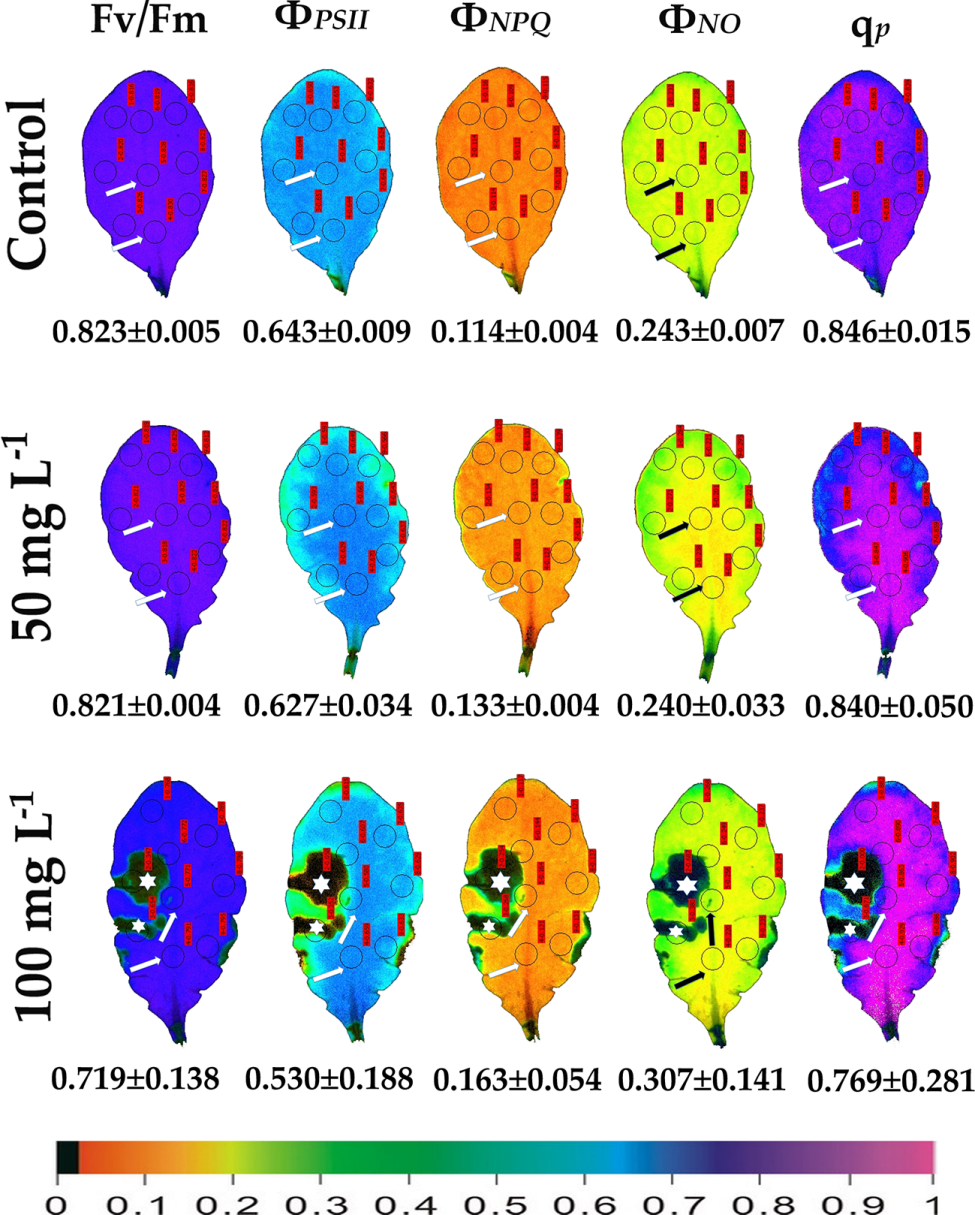
Color-coded images of the chlorophyll fluorescence parameters F*v*/F*m* acquired after dark adaptation, and of Φ*_PSII_*, Φ*_NPQ_*, Φ*_NO_*, and *q* _p_, acquired with 140 μmol photons m^–2^ s^−1^ light intensity, after exposure to 0 (control), 50 and 100 mg L^−1^ BPA for 6 h. The color code depicted at the bottom of the images ranges from black (pixel values 0.0) to purple (1.0). Nine areas of interest (AOIs) are shown in each image together with the average value (± SD) of the whole leaf for each photosynthetic parameter. Arrows in the images point at the mid vein AOIs that were not affected or affected (negatively or positively) by the BPA application. Asterisks on the images note the AOIs that were negatively affected by the BPA application.

**Figure 2:**
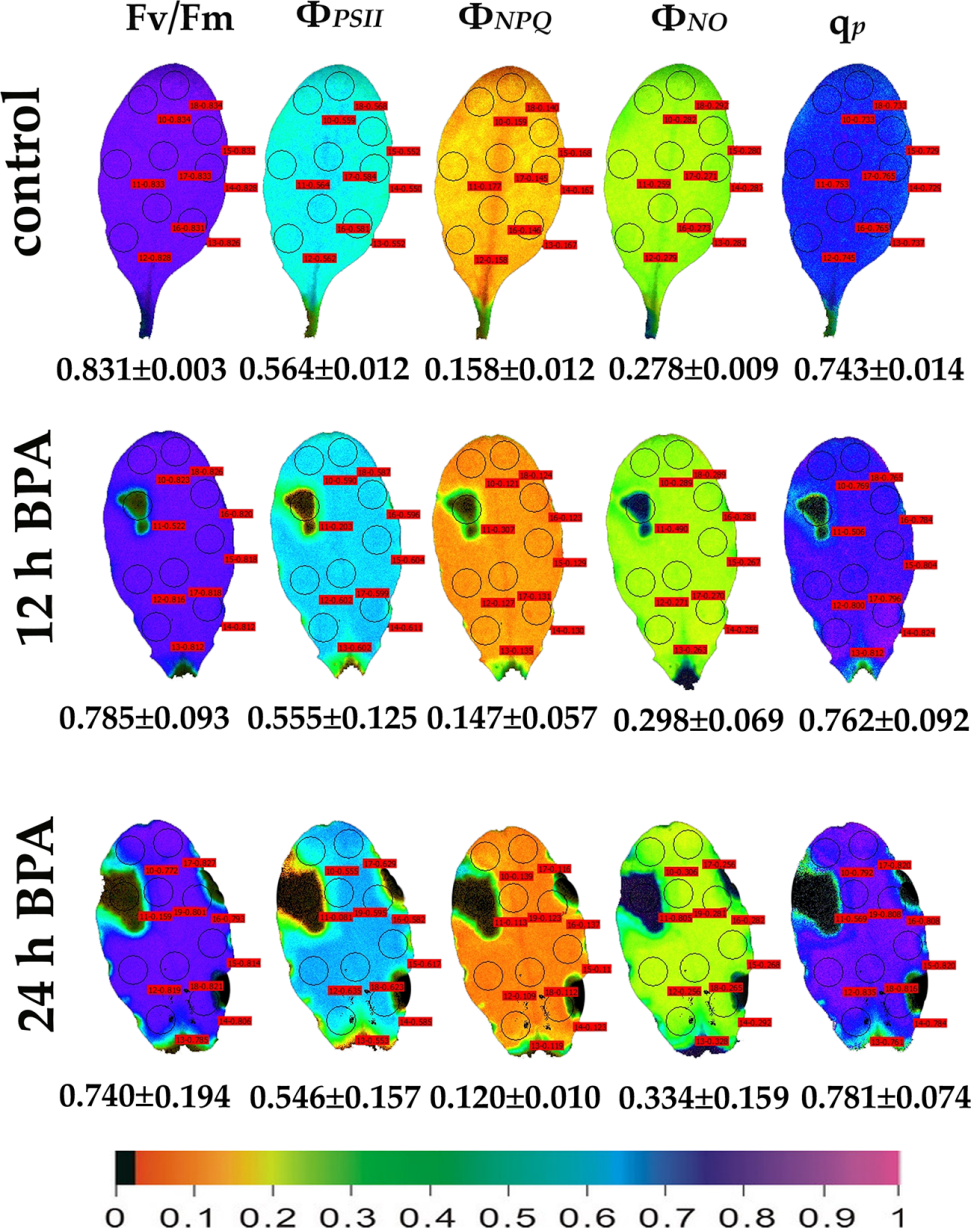
Color-coded images of the chlorophyll fluorescence parameters F*v*/F*m* acquired after dark adaptation and of Φ*_PSII_*, Φ*_NPQ_*, Φ*_NO_*, and *q* _p_, acquired with 140 μmol photons m^−2^ s^−1^ light intensity, after exposure to 0 (control) and 50 mg L^−1^ BPA for 12 and 24 h. The color code depicted at the bottom of the images ranges from black (pixel values 0.0) to purple (1.0). Nine or ten AOIs are shown in each image together with the average value (± SD) of the whole leaf for each photosynthetic parameter.

**Figure 3:**
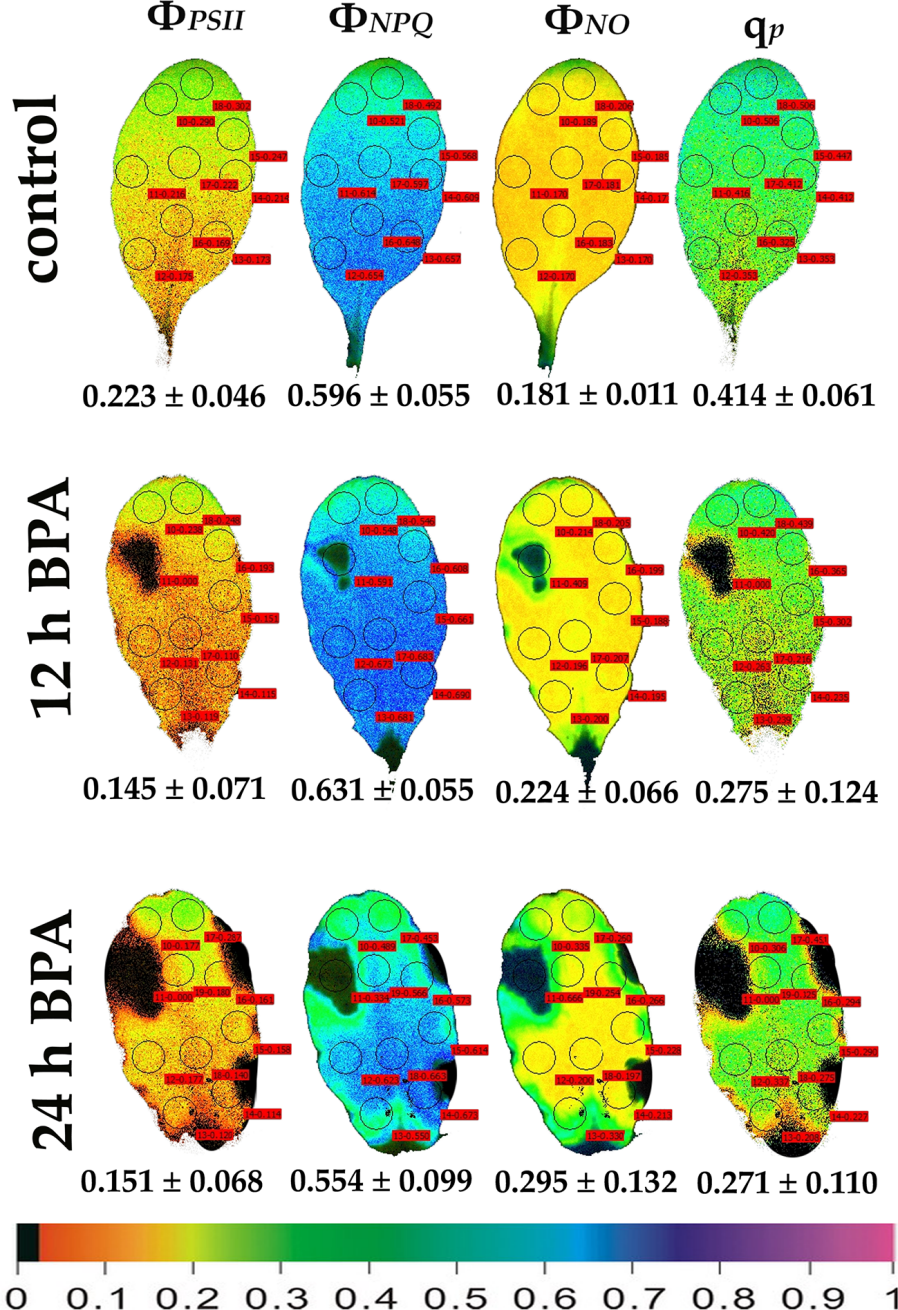
Color-coded images of the chlorophyll fluorescence parameters Φ*_PSII_*, Φ*_NPQ_*, Φ*_NO_*, and *q* _p_, acquired with 1000 μmol photons m^−2^ s^−1^ light intensity, after exposure to 0 (control) and 50 mg L^−1^ BPA for 12 and 24 h. The color code depicted at the bottom of the images ranges from black (pixel values 0.0) to purple (1.0). Nine or ten AOIs are shown in each image together with the average value (± SD) of the whole leaf for each photosynthetic parameter.

We evaluated the effects of 50 and 100 mg L^−1^ BPA treatments for 6, 12 and 24 h on the chlorophyll fluorescence parameters F*v*/F*m*, Φ_PSII_, Φ_NPQ_, Φ_NO_, and q_p_ in order to evaluate BPA effects on PSII functionality. Color-coded images after 20 min dark adaptation of F*v*/F*m*, and after 5 min illumination (140 μmol photons m^−2^ s^−1^) for Φ_PSII_, Φ_NPQ_, Φ_NO_, and q_p_, of either control (leaves maintained in Petri dishes on soaked filter paper with distilled water) or of BPA treated leaves (maintained in Petri dishes on soaked filter paper with 50 and 100 mg L^−1^ aqueous BPA solution) for 6 h are presented in **Figure 1**. We observed a spot-like mode of action of BPA after 6 h treatment with 100 mg L^−1^ that could not be observed after 6 h treatment with 50 mg L^−1^ (**Figure 1**). However, the spot-like mode of BPA action was visible after 12 and 24 h treatment with 50 mg L^−1^ under both low light (**Figure 2**) and high light (**Figure 3**) intensities.

After 6 h treatment with 50 mg L^−1^ BPA Φ_PSII_ values of the mid vein AOIs (arrows) increased compared to their corresponding controls, while Φ_NO_ values of the mid vein AOIs decreased compared to their corresponding controls (**Figure 1**). In addition, the fraction of open PSII reaction centers (q_p_) of the mid vein AOIs (arrows) increased, compared to their corresponding controls (**Figure 1**). After 6 h treatment with 100 mg L^−1^ BPA, the same pattern as 50 mg L^−1^ BPA treatment was observed for Φ_PSII_ and Φ_NO_ values but only for the lower mid vein AOI, while q_p_ values increased for both the mid vein AOIs (arrows) (**Figure 1**). This treatment (100 mg L^−1^ BPA, 6 h) decreased significantly F*v*/F*m* value of the whole leaf and mid vein AOIs (arrows) compared to the control values (**Figure 1**).

Under 12 and 24 h treatments with 50 mg L^−1^ BPA the fraction of open PSII reaction centers (q_p_) of the whole leaf increased (with the exception of the spot like affected AOI) compared to control (**Figure 2**). Exposure to high light (1000 μmol photons m^−2^ s^−1^) of *Arabidopsis* leaves resulted in increased leaf heterogeneity under non-stressed conditions of the chlorophyll fluorescence parameters Φ_PSII_, Φ_NPQ_, and q_p_ as was evidenced from the whole leaf color-coded images and the increased standard deviation (**Figure 3**). After 12 h treatment with 50 mg L^−1^ BPA, whole leaf Φ_PSII_ value under high light decreased significantly, with the spot like affected AOI to have Φ_PSII_ value 0, and all the reaction centers (q_p_) closed (**Figure 3**).

**Figure 4:**
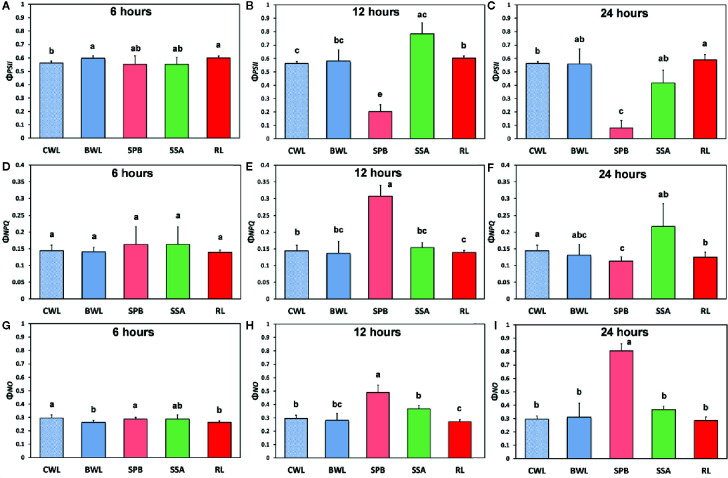
The effects of 50 mg L^−1^ BPA on the effective quantum yield of photochemical energy conversion in PSII (Φ*_PSII_*) after 6-h **(A)**, 12-h **(B),** and 24-h exposures **(C);** the quantum yield of regulated non-photochemical energy loss in PSII (Φ*_NPQ_*) after 6 h **(D)**, 12** h (E)** and 24** h** exposure **(F);** and the quantum yield of non-regulated energy loss in PSII (Φ*_NO_*) after 6-h **(G)**, 12-h **(H),** and 24-h exposures **(I)**, all measured at 140 μmol photons m^−2^ s^−1^ in *Arabidopsis thaliana* leaves. Symbol explanation: Control Whole Leaves (CWL) maintained in Petri dishes on filter paper soaked with distilled water and considered as controls; BPA treated whole leaves (BWL) maintained in Petri dishes on filter paper soaked with 50 mg L^−1^ BPA; spot BPA zone (SPB) the spot like zone that was affected by BPA; spot surrounding area (SSA); Rest of the Leaf (RL), that is the leaf area that remains if the Spot BPA zone (SPB) and the spot surrounding area (SSA) are subtracted from the BPA-treated Whole Leaves (BWL). Error bars on columns are standard deviations based on three independent biological replicates under all treatments. Columns under the same time treatment with the same letter are statistically not different (*P* < 0.05).

The effects of BPA treatment on the allocation of the absorbed light energy in *A. thaliana* leaves are presented in **Figure 4**. We estimated the fraction of the absorbed light energy that is used for photochemistry (Φ*_PSII_*) (**Figures 4A–C**), the fraction that is lost by regulated heat dissipation (Φ*_NPQ_*) (**Figures 4D–F**), and the fraction of non-regulated energy loss (Φ*_NO_*) (**Figures 4G–I**). These three quantum yields (Φ*_PSII_*, Φ*_NPQ_*, and Φ*_NO_*) add up to unity. After 6 h treatment with 50 mg L^−1^, Φ_PSII_ values of BPA-treated whole leaves (BWL) increased 6% compared to control (CWL), without any significant difference at the BPA zone (SPB) and the surrounding area (SSA) compared to CWL, but with a significant increased (7%) value at the rest of the leaf (RL) (**Figure 4A**). After 12 and 24 h treatment, Φ_PSII_ values at the spot BPA zone (SPB) decreased (64% and 86% respectively), while at the rest of the leaf (RL) increased (7% and 5% respectively) compared to CWL (**Figures 4B, C**, respectively). Φ_NPQ_ values did not change after 6 h treatment with 50 mg L^−1^ BPA at all evaluated zones (**Figure 4D**), but increased significantly (114%) at the SPB after 12 h treatment (**Figure 4E**), while decreased significantly (21%) at the same zone after 24 h treatment compared to CWL (**Figure 4F**). Φ_NO_ values did not change after 6 h treatment with 50 mg L^−1^ at the spot BPA zone (SPB) compared to CWL (**Figure 4G**), but after 12 and 24 h treatment increased significantly (66% and 173% respectively) at the same zone compared to CWL (**Figures 4H, I**, respectively). At the rest of the leaf (RL) after 12 h treatment Φ_NO_ decreased (8%) (**Figure 4H**), while after 24 h treatment remained unchanged (**Figure 4I**) compared to CWL.

**Figure 5:**
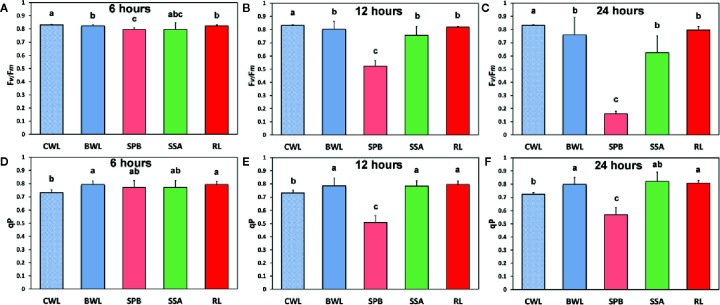
The effects of 50 mg L^−1^ BPA on the maximum photochemical efficiency (F*v*/F*m*) after 6-h **(A)**, 12-h **(B),** and 24-h exposure **(C)**, and the redox state of the plastoquinone (PQ) pool (q_p_), a measure of the fraction of open PSII reaction centers after 6-h **(D)**, 12-h **(E),** and 24-h exposures **(F)**, all measured at 140 μmol photons m^−2^ s^−1^ in *Arabidopsis thaliana* leaves. Symbol explanation: control whole leaves (CWL) maintained in Petri dishes on filter paper soaked with distilled water and considered as controls; BPA-treated whole leaves (BWL) maintained in Petri dishes on filter paper soaked with 50 mg L^−1^ BPA; spot BPA zone (SPB) the spot like zone that was affected by BPA; spot surrounding area (SSA); rest of the leaf (RL), that is the leaf area that remains if the spot BPA zone (SPB) and the spot surrounding area (SSA) are subtracted from the BPA-treated whole leaves (BWL). Error bars on columns are standard deviations based on three independent biological replicates under all treatments. Columns under the same time treatment with the same letter are statistically not different (*P* < 0.05).

The maximum photochemical efficiency (F*v*/F*m*) was the only chlorophyll fluorescence parameter that was negatively affected in a dose- and time-dependent manner in the BPA-treated whole leaves (BWL) and not only at the spot BPA zone (SPB) (**Figures 5A–C**), as was observed at the other measured parameters (**Figure 4**). The redox state of the plastoquinone (PQ) pool (q_p_), a measure of the fraction of open PSII reaction centers, increased after 6, 12 and 24 h treatment in BPA-treated whole leaves (BWL) and the rest of the leaf (RL) (**Figures 5D–F**).

**Figure 6:**
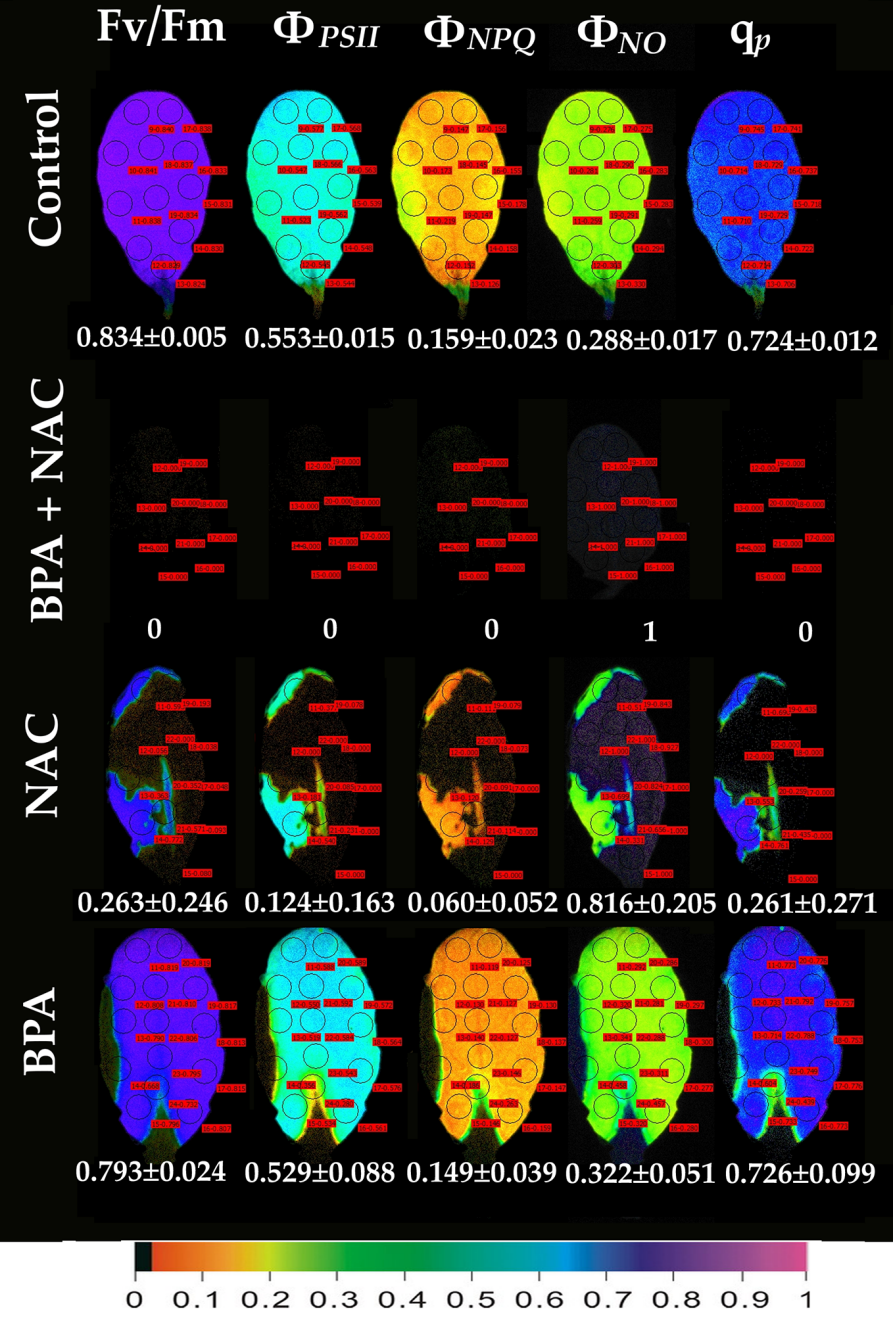
Color-coded images of the chlorophyll fluorescence parameters F*v*/F*m*, Φ*_PSII_*, Φ*_NPQ_*, Φ*_NO_*, and *q* _p_, after exposure of *Arabidopsis thaliana* leaves to 0 (control), or 50 mg L^−1^ BPA plus 500 µM NAC, or 500 µM NAC alone, or only 50 mg L^−1^ BPA, for 24 h. The color code depicted at the bottom of the images ranges from black (pixel values 0.0) to purple (1.0). Ten to fourteen AOIs are shown in each image together with the average value (± SD) of the whole leaf for each photosynthetic parameter.

Exposure of *A. thaliana* leaves to 50 mg L^−1^ BPA plus 500 µM NAC for 24 h eliminated whole leaf Φ_PSII_ and Φ_NPQ_ having as a consequence only Φ_NO_ to occur, and all the reaction centers to be closed (**Figure 6**). Exposure of leaves to 500 µM NAC alone for 24 h resulted in milder effects on chlorophyll fluorescence parameters (**Figure 6**). However, a 68% reduction of the maximum photochemical efficiency (F*v*/F*m*), a 78% reduction in the absorbed PSII light that is used for photochemistry (Φ_PSII_) and a 62% reduction in the photoprotective energy dissipation as heat (Φ_NPQ_) occurred. As a result, a 2.8-fold increase in the quantum yield of non-regulated energy loss in PSII (Φ_NO_) occurred and a 64% reduction in the fraction of open PSII reaction centers (q_p_) (**Figure 6**). Twenty-four h treatment with 50 mg L^−1^ BPA alone had milder effects on chlorophyll fluorescence parameters from all the treatments (**Figure 6**).

**Figure 7:**
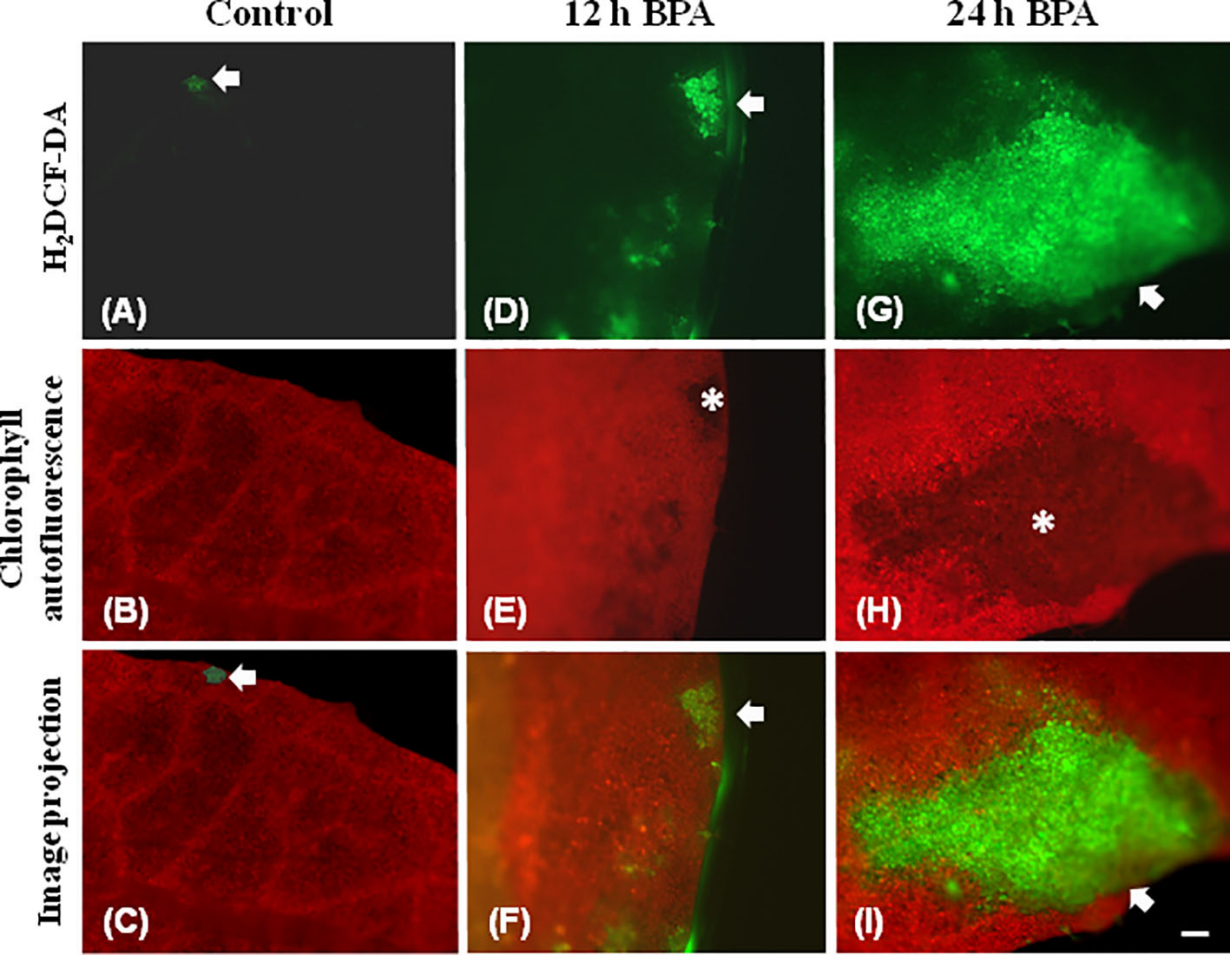
Chlorophyll autofluorescence images (red) and after H_2_DCF-DA staining, indicating H_2_O_2_ production (green) in untreated leaves (control) **(A–C)**, 12 h **(D–F)**, and 24 h **(G–I)** exposure to 50 mg L^−1^ BPA. In untreated leaves **(A–C)** very weak H_2_DCF-DA staining was detected (**A, C**, arrows), while leaf margin areas exhibited strong chlorophyll autofluorescence **(B)**. BPA-treated leaves exhibit reduced chlorophyll autofluorescence areas **(E, H**, asterisks) that coincide with time-dependent increased production of H_2_O_2_ (arrows in **D, F, G, I**). Bar, 100 μm.

Leaf spot BPA areas, negatively affected by BPA (asterisks in **Figure 1** and **Figures 2**, **3**, **6**), showed highly increased chlorophyll fluorescence heterogeneity compared to the rest leaf, with a reduced fraction of open PSII reaction centers (q_p_) and an increased non–regulated energy loss (*Φ*_NO_). In those areas chlorophyll autofluorescence loss coexisted with an increased H_2_O_2_ production as was shown after H_2_DCFDA staining (**Figures 7D–I**), a phenomenon not present in control leaves stained with H_2_DCFDA (**Figures 7A–C**).

## Discussion

ROS production (especially H_2_O_2_) stimulated by BPA has been linked with the PSII photoinhibition observed under BPA treatments (Qui et al., 2013; Li Y. T. et al., 2018). BPA seems to affect the electron transport between PSII and PSI (Qiu et al., 2013), but it does not exert a direct PSII damage (Li Y. T. et al., 2018), since the block of ETR by BPA under high light is attributed to CO_2_ fixation inhibition (Li Y. T. et al., 2018). Moreover, in soybean BPA-treated seedlings, the inhibition of growth was related to the decrease in photosynthesis due to a decrease in the content of chlorophyll, the net photosynthetic rate and changes in the chlorophyll fluorescence parameters F*v*/F*m*, Φ_PSII_, and ETR that were decreased, compared to the control (Qiu et al., 2013). In the present study, under low light, PSII function was negatively influenced only at the spot affected BPA zone in a dose- and time-dependent manner, while at the whole leaf only the maximum photochemical efficiency (F*v*/F*m*) was negatively affected (**Figures 4**, **5**). This BPA induced decrease in F*v*/F*m* suggests photoinhibition of PSII caused by ROS through inhibition of CO_2_ assimilation and over-reduction of ETR that increased ROS (H_2_O_2_) generation inhibiting the repair of photodamaged PSII (Li Y. T. et al., 2018). In our experiments, under high light all PSII photosynthetic parameters (Φ*_PSII_*, Φ*_NPQ_*, Φ*_NO_*, and *q* _p_) were negatively affected by BPA application, in a time-dependent manner (**Figure 3**), also in detached leaves directly exposed to BPA aqueous solutions. Increased BPA concentration (100 mg L^−1^ BPA, 6 h exposure *q* _p_ image, **Figure 1**) or high light exposure (50 mg L^−1^ BPA, 12 and 24 h exposure *q* _p_ images, **Figure 3**) resulted to necrotic death-like spots in leaves, probably caused by increased H_2_O_2_ accumulation visible in a spot-like manner (**Figure 7**).

Using the H_2_DCFDA staining we observed an increased H_2_O_2_ accumulation, in spots in the leaf periphery (**Figure 7**) under BPA treatments. BPA-induced H_2_O_2_, could be a toxic ROS able to cause damage to a variety of cellular structures but in parallel can act as a potent signaling molecule involved in BPA stress response, as it has been demonstrated in a plethora of physiological functions (Foyer and Shigeoka, 2011; Petrov and Breusegem, 2012; Foyer and Noctor, 2013; Moustaka et al., 2015; Moustakas et al., 2016). H_2_O_2_ enters the cell through aquaporins and regulates physiological and cellular processes (Foyer, 2020; Wu et al., 2020). Still, H_2_O_2_ can diffuse through leaf veins to act as a long-distance regulator molecule activating the antioxidant defense during stress in plants (Wilson et al., 2006; Mittler et al., 2011; Petrov and Breusegem, 2012; Moustaka et al., 2015; Moustakas et al., 2017; Antonoglou et al., 2018; Sperdouli et al., 2019). Since H_2_O_2_ travels through veins faster than from cell to cell, it seems logic why at 6 h treatments with 50 and 100 mg L^−1^ BPA the fraction of open PSII reaction centers (q_p_) of the mid veins AOIs (arrows) were those areas that increased first, compared to their corresponding controls (**Figure 1**), while just at longer treatments (12 and 24 h with 50 mg L^−1^ BPA) whole leaf q_p_ values increased (with the exception of the spot like affected AOIs) compared to controls (**Figure 2**). However, the exposure of *A. thaliana* leaves to high light and 50 mg L^−1^ BPA (12 and 24 h treatment) (**Figure 3**) decreased the effective quantum yield of PSII (Φ_PSII_) and over-reduced the redox state of PQ pool closing a fraction of open PSII reaction centers (q_p_) (Murabakshina et al., 2010; Foyer and Shigeoka, 2011; Mignolet-Spuyt et al., 2016; Moustaka et al., 2018). In agreement to our results, Li et al. (2018b) have noticed that, under high light the BPA treatment changed similarly Φ_PSII_ and q_p_, and concluded that the decrease in Φ_PSII_ was mainly due to the decline in q_p_ rather than to the decrease in the efficiency of open PSII centers to utilize the absorbed light (F*v*/F*m*).

The spatiotemporal pattern of BPA effects on *A. thaliana* treated leaves points out to the differential defense response of each cell to BPA stress as it has been shown for other abiotic stress factors, e.g., drought (Sperdouli and Moustakas, 2012), hypoxia (Stasolla et al., 2019), paraquat (Moustakas et al., 2016), and heavy metals as Zn (Moustakas et al., 2019a), or Cd (Moustakas et al., 2019b). This can be due to the fact that plant cells have to defend themselves independently since they lack specialized cells and effective plant defense strongly relies in each single cell (Ruano and Scheuring, 2020).

In an earlier study, BPA residual concentrations had a negative correlation with H_2_O_2_ levels, i.e., an increase in H_2_O_2_ seemed to reduce BPA levels inside the plant tissue (Zhang et al., 2018). These results allowed to speculate that BPA could either be a direct target of ROS, and therefore subjected to oxidation (Reis et al., 2014) or ROS molecules could activate a cascade of secondary metabolic reactions degrading BPA (Noureddin et al., 2004) and finally the ROS-activated antioxidant enzymes could destroy BPA (Kang and Kondo, 2006). So in soybean roots, H_2_O_2_ initiated accumulation offered a protection against BPA (Zhang et al., 2018). Likewise, in our experimental system if BPA-induced H_2_O_2_ accumulation (**Figure 7**) is hindered, with NAC application (**Figure 6**), leaf photosynthesis is utterly being interrupted (*Φ*_NO_=1; **Figure 6**). Therefore, this H_2_O_2_ production could be necessary for promoting signaling events that could assist the plant to alleviate BPA-stress. NAC is a strong ROS scavenger (Zafarullah et al., 2003) since the SH group of NAC is able to donate an H-atom or an electron. Numerous researchers have used it as a mean to reduce either the stress-induced or naturally occurring H_2_O_2_ (Livanos et al., 2012; Muranaka et al., 2013; Sun et al., 2014; Livanos et al., 2016; Adamakis and Eleftheriou, 2019; Colak et al., 2019). Generally, NAC is being considered not toxic for plants and the environment even when applied in high concentrations for large periods of time (i.e., Sun et al., 2014), able to alleviate oxidative stress induced by several stressors, e.g., heavy metals (Sun et al., 2014; Colak et al., 2019). However, when used to diminish naturally occurring ROS several cellular defects have been noticed. For instance, when NAC was applied in wheat or *A. thaliana* roots, microtubule organization was affected (Livanos et al., 2012) while cytokinesis failed to be accomplished (Livanos et al., 2016). The above indicated that ROS is an important factor enrolled in the microtubule assembly and cell division completion (Livanos et al., 2012; Livanos et al., 2016). Expanding the beneficial role of both naturally occurring and BPA-induced H_2_O_2_, we here noticed that ROS seem to have also pivotal role in the light reactions of photosynthesis. This comes as no surprise since the electron transport between PSII and PSI is a major source of ROS, which are considered more as signaling molecules rather than damaging ones (Hajiboland, 2014; Foyer et al., 2017). The role of chloroplast antioxidants, that often have overlying or interrelating functions, is not to totally eliminate O_2_^• –^, H_2_O_2_ and ^1^O_2_, but rather to achieve an appropriate balance between production and subtraction so that to match with the operation of photosynthesis and permit an efficient spread of signals to the nucleus (Foyer, 2018). When NAC diminished these naturally occurring ROS (**Figure 6**), all of the PSII photosynthetic parameters (Φ*_PSII_*, Φ*_NPQ_*, Φ*_NO_*, and *q* _p_) were severely affected, indicating the importance of naturally occurring ROS in PSII photochemistry. Now it is well established that ROS are a necessary part of subcellular and intercellular communication in plants and that some of their signaling functions require ROS-metabolizing systems (Noctor et al., 2018).

Our results confirm the view that ROS-removing systems are considering ROS as beneficial molecules that regulate damaging ROS below dangerous levels (Noctor et al., 2018). So, one can easily conclude, that ROS seem to play a pivotal role in plant response against BPA toxicity (Zhang et al., 2018), as we observed in BPA-affected leaves of *A. thaliana.* While the concept that animal and plant cells need to remove ROS production to avoid extreme and permanent oxidation was the dominant view in the literature, the opinion is now shifting towards recognition of a positive role of ROS as well (Noctor and Foyer, 2016; Foyer et al., 2017). ROS generation can activate the plant’s defense mechanisms in order to cope with the oxidative stress damage and are essential for redox sensing, signaling and regulation (Petrov et al., 2015; Foyer, 2018; Malea et al., 2019). Plants have developed during the course of evolution numerous ROS-generating pathways tightly accomplishing plant function and development (Noctor et al., 2018). Therefore, the necrotic death-like spots under BPA exposure could be due to ROS accumulation, but H_2_O_2_ production has dual function in plants playing also a protective role in BPA-induced stress. A crucial ROS role in the photochemical reactions of photosynthesis is further on confirmed.

## Data Availability Statement

All datasets presented in this study are included in the article/supplementary material.

## Author Contributions

I-DA and MM conceived and designed the experiments, I-DA and IS performed the experiments, I-DA, EE, and MM wrote the original draft of the manuscript. All authors contributed to the article and approved the submitted version.

## Conflict of Interest

The authors declare that the research was conducted in the absence of any commercial or financial relationships that could be construed as a potential conflict of interest.
